# Unleashing the potential of mRNA: Overcoming delivery challenges with nanoparticles

**DOI:** 10.1002/btm2.10713

**Published:** 2024-08-15

**Authors:** Qiang Chen, Ku‐Geng Huo, Sheng‐Min Ji, Shu‐De Pang, Tian‐Ying Sun, Yi Niu, Zi‐Hao Jiang, Peng Zhang, Shu‐Xin Han, Jin‐Yao Li

**Affiliations:** ^1^ Xinjiang Key Laboratory of Biological Resources and Genetic Engineering College of Life Science & Technology, Xinjiang University Urumqi China; ^2^ Cyagen Biosciences (Guangzhou) Inc. Guangzhou Guangdong China; ^3^ School of Pharmacy, Key Laboratory of Molecular Pharmacology and Drug Evaluation, Ministry of Education, Collaborative Innovation Center of Advanced Drug Delivery System and Biotech Drugs in Universities of Shandong Yantai University Yantai China; ^4^ Department of Pharmaceutics School of Pharmacy, Qingdao University Qingdao China

**Keywords:** delivery strategy, mRNA, nanomaterial, nanotechnology

## Abstract

Messenger RNA (mRNA) has emerged as a promising therapeutic strategy for various diseases, including cancer, infectious diseases, and genetic disorders. The mRNA‐based therapeutics have gained significant attention due to their ability to regulate targeted cells, activate immune cells, and avoid potential risks associated with DNA‐based technology. However, the clinical application of mRNA in cancer therapy is hindered by the instability of RNA, physiological barriers, and the risk of immunogenic hurdles. To overcome these challenges and ensure the safe delivery of mRNA therapeutics to target sites, nanoparticle‐based delivery systems have been explored as potential tools in vitro and in vivo applications. This review provides a comprehensive overview of the current status of mRNA therapy, discussing its advantages and limitations, delivery strategies and materials, as well as applications in different fields. By exploring these aspects, the researcher can gain a more complete understanding of the current state, prospects, and challenges of mRNA technologies.

**Abbreviations**
AAV: adeno‐associated virus
AdV: adenovirus
APC: antigen‐presenting cells
BCR: B cell receptor
CPs: cationic polymers
CS: chitosan
DCs: dendritic cells
EUA: emergency use authorization
eVLP: engineered virus‐like particle
HA: hyaluronic acid
IFN‐α: interferon alpha
LNP: lipid nanoparticles
LV: lentivirus
MHC: histocompatibility complexes
mRNA: messenger RNA
PAMAM: dendritic polyamide
PEG: polyethylene glycol
PEI: polyethyleneimine
PICs: polyelectrolyte complexes
PLL: polylysine
PPI: polyimide
RV: retrovirus
TLRs: Toll‐like receptors
TNF‐α: tumor necrosis factor‐alpha
VLPs: virus‐like particles
β‐CD: β‐Cyclodextrin

## 1 INTRODUCTION

**FIGURE 1:**
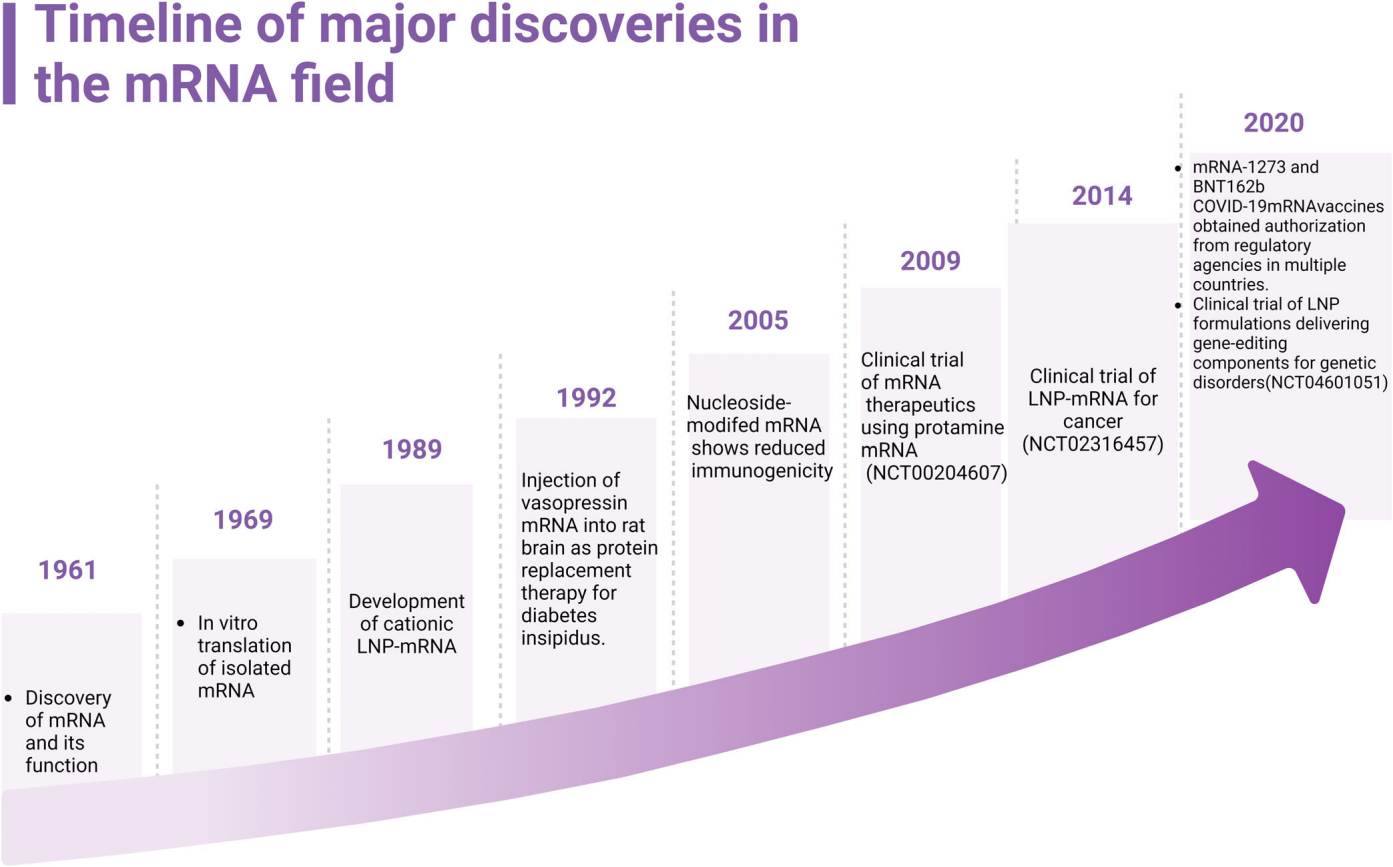
Timeline of major discoveries in the mRNA field. Created with BioRender.com.

The groundbreaking discovery of messenger RNA (mRNA) by François Jacob and Jacques Monod in 1961 and the fundamental understanding of its machinery and functions mark the beginning of mRNA‐based therapy, which has been used in a wide range of disease areas including cancers, genetic disorders, diabetes, inflammatory diseases, and neurodegenerative diseases.^1^, ^2^, ^3^, ^4^, ^5^ The first attempt to use delivery exogenous mRNAs to produce functional proteins was performed by Wolff et al. in 1990 expressing simple reporters in mouse muscle.^6^ The 21st century ushered in a golden age for mRNA research. Researchers are primarily interested in understanding the challenges and opportunities associated with the current mRNA delivery nano‐platform. For instance, achieving effective and safe mRNA therapy in vivo requires an efficient and safe delivery system to transport therapeutic genes to relevant organs or tissues in the body. This is a major challenge for current in vivo gene therapy plans. Nano‐technological advancements, including increased mRNA stability, lowered immunogenicity, and improved delivery efficiency, propelled the field forward. The results of the first clinical trial of mRNA therapeutics were published by Weide et al. in 2008 using mRNA vaccine for the treatment of metastatic melanoma. However, clinical effectiveness was not observed.^7^ In 2017, CureVac for the first time demonstrated a functional anybody boost against the viral antigen by an mRNA vaccine, highlighting the potential of mRNA technology in vaccine development.^8^ The COVID‐19 pandemic thrust mRNA therapy to clinical use. Two mRNA vaccines, developed by Pfizer‐BioNTech and Moderna, respectively, received emergency use authorization (EUA) and played a pivotal role in curbing the spread of the virus,^9^, ^10^ but their long‐term safety remains to be determined (profile extensively reviewed elsewhere^11^) (Figure 1).

**FIGURE 2:**
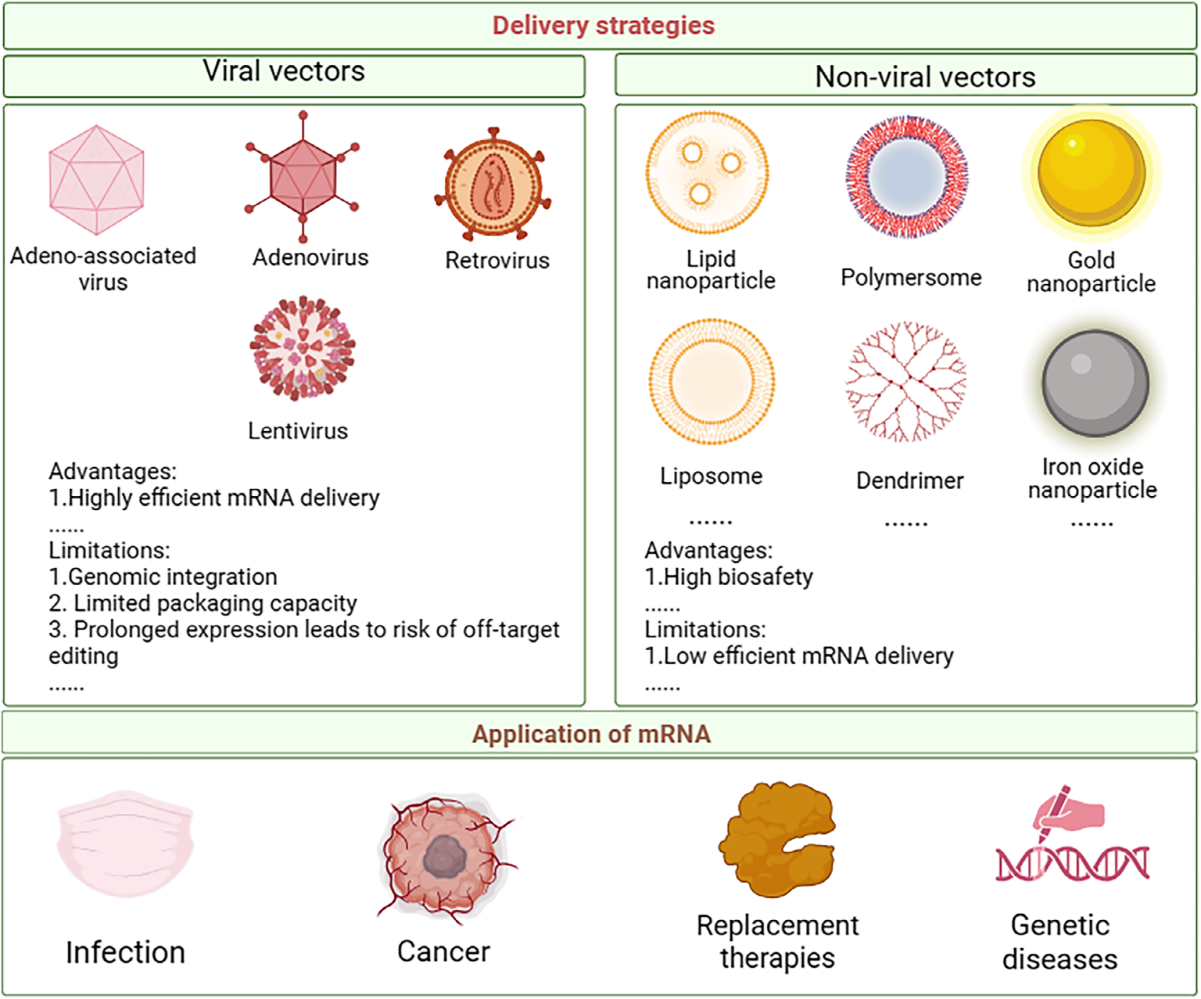
An overview of delivery strategies and applications of mRNA. Various viral and non‐viral vectors can be used to deliver nucleic acids. mRNA technology is a powerful tool for various biomedical applications, including cancer and viral infections, replacement therapies for therapeutic proteins, and therapies for genetic diseases. Created with BioRender.com.

Unlike DNA‐based technology for protein expression, mRNA does not infect the original genome as it does not need to enter the nucleus to carry out its functions.^12^, ^13^ When used as a vaccine, mRNA can activate immune cells to release cytokines, such as tumor necrosis factor‐alpha (TNF‐α) and interferon alpha (IFN‐α), which enhance the immune effects of the expressed antigens.^9^, ^14^ However, the clinical application of mRNA in cancer therapy is hindered by the instability of RNA and the presence of physiological barriers that inhibit the delivery and transfection of RNA.^1^, ^15^, ^16^ In addition, exogenous RNA is more likely to be cleared by the body's defense systems, including exonucleases and RNases that are responsible for RNA degradation.^4^, ^12^, ^17^ Moreover, mRNA delivery to the target cell efficiently is still a challenging task.^12^ To overcome these immunogenic hurdles and ensure the safe delivery of mRNA therapeutics to the target sites, nanoparticle‐based delivery systems have been explored as potential tools for in vitro and in vivo applications. Here, we provide a comprehensive review of the current status of mRNA therapy, discussing its advantages and limitations, delivery strategies and materials, as well as applications in different fields (Figure 2).

## 2 THE CURRENT SITUATION OF GENE DELIVERY

In 1989, Rosenberg et al. conducted a pioneering human gene therapy experiment using retroviral vectors for the treatment of advanced melanoma. Since then, ample studies have showcased the immense potential of gene delivery for treating and preventing a wide range of human diseases.^18^ In recent years, numerous gene delivery systems have emerged, which can typically be categorized into viral and non‐viral vectors.^19^ Viral vectors typically deliver DNA into the target cells. The exogenous DNA will then enter the nucleus of the target cells and express the transgene. Non‐viral vectors including lipid nanoparticles, polymer nanoparticles, and inorganic nanoparticles, on the other hand, can directly package and deliver mRNA.^20^, ^21^, ^22^ Delivering directly mRNA offers several advantages over‐delivering DNA. It does not need to enter the cell nucleus to function, therefore exhibiting exhibit higher transient expression,^23^ and reducing the risk of creating insertional mutagenesis.^5^, ^24^ Additionally, mRNA has a transient effect, making it easier to regulate the amount of protein produced during therapy while reducing the risk of long‐lasting side effects.^25^ mRNA therapeutic strategies are highly cost‐effective and easy to mass‐produce due to their simplicity.^5^ It is noteworthy, however, that mRNA therapy also has certain limitations, especially when it comes to certain applications. The mRNA is more susceptible to degradation. Meanwhile, like cancer vaccines, mRNA vaccines also exhibit increased inflammation and a higher incidence of adverse effects compared with DNA vaccines.^26^

Currently, gene therapy and vaccine development using DNA and mRNA present challenges to drug delivery, including phagocytosis, enzymatic degradation, protein absorption, non‐specific immunogenesis, and cellular internalization barriers.^27^, ^28^ Vectors are delivery systems that can overcome these obstacles. They protect genes from degradation and facilitate gene transcription.^27^, ^29^

### 2.1 Virus vectors

The researchers first utilized the characteristics of the virus for DNA delivery. Different viral vectors have their unique advantages and limitations. Adeno‐associated virus (AAV) is non‐pathogenic in humans and rarely causes genome integration and severe immune responses. It can achieve long‐term expression of the transgene that can last for up to years, which makes it a perfect gene therapy candidate for hereditary diseases. However, it has a limited packaging capacity of only ~4.8 kb, and the development of anti‐AAV neutralizing antibodies strongly limits repeated administration.^30^, ^31^, ^32^ Adenovirus (AdV) has a larger packaging capacity (up to 8 kb) but can cause a stronger immune response, and the transgene expression is typically transient, making it a better candidate for vaccine development.^33^, ^34^ Lentivirus (LV) and retrovirus (RV) can accommodate even larger genes. However, genomic integration limits their use to ex vivo applications such as CAR‐T cell therapy.^35^ In summary, viral vectors have shown great potential in delivering gene‐edited drugs in current clinical and preclinical studies. Due to the strong transfection efficiency and drug delivery ability of viral vectors in various cell types, high gene editing efficiency has been observed in various organs. Viral vectors have significant limitations, including immunogenicity, long‐term expression of gene‐edited drugs, non‐targeted gene editing, the possibility of genome integration, manufacturing costs, and dose‐limited toxicity.

### 2.2 Non‐viral vectors

Non‐viral vectors effectively overcome the limitations of viral vectors. They can directly deliver mRNA to avoid the risk of gene integration caused by viral vectors delivering DNA. In addition, Synthetic vectors are comparatively easier to synthesize and able to deliver larger genetic payloads than viral vectors. Non‐viral vectors also allow repeated administration and offer a greater safety profile since they are less immunogenic and not insertional.^36^, ^37^, ^38^ Consequently, mRNA‐based therapies delivered by non‐viral vectors have gained growing interest as a safe and effective method for clinical applications such as protein replacement,^39^ vaccination,^9^ tumor immunotherapy,^40^ and genome engineering.^41^

## 3 METHODS OF MRNA DELIVERY

The use of mRNA gene therapy has the potential to decrease the risk of gene integration. In the organism, the stability of mRNA can be enhanced by modifying the structure of the 5′‐terminal (Cap and UTRs) and 3′‐terminal (UTRs and Ploy(A)), thereby extending its half‐life.^12^, ^42^ Currently, most mRNA products have a synthetic UTR sequence from α‐globin or β‐globin,^43^ but UTR optimization can improve protein expression by a few folds.^44^ Screening and customizing future UTR sequences for the target cell and disease microenvironment could optimize protein synthesis per‐mRNA transcript.^45^, ^46^ Furthermore, Circular RNAs (circRNAs) are a class of naturally occurring or synthetic closed RNA molecules that lack 5′ and 3′ ends.^47^, ^48^ In contrast, their distinctive covalently closed structures prevent RNA degradation by exonucleases, thereby conferring them with high pharmaceutical stability and biostability relative to the current standard of care, linear mRNA.^49^ Natural circRNAs can be classified as either non‐coding RNAs or protein‐coding RNAs. The latter category was recently discovered.^49^ In recent times, synthetic circRNA has been developed to explore its potential applications as a novel class of mRNA therapeutics and vaccines.^49^ The extended longevity of circRNA increases total protein yield without increasing protein expression amplitude compared with linear modified mRNA.^50^, ^51^, ^52^ This eliminates the need for costly 5′ capping and cumbersome 3′ poly(A) tail by introducing internal ribosomal entry site (IRES) sequences.^44^, ^52^ Furthermore, circularization strongly reduces RIG‐1 and Toll‐like receptor recognition without using chemical substitution.^51^ Researchers could explore developing replicon circRNA vaccines to enhance RNA stability. Although in vivo delivery of circRNAs in mice has shown promise, the issue of targeting specific tissues remains unsolved.^53^ Self‐amplifying mRNAs (saRNAs) exploit the replication mechanism of RNA alphaviruses to amplify transcripts in the cytoplasm, but replace the viral structural genes with the gene of interest.^44^, ^54^, ^55^ saRNA transcripts enable prolonged‐expression kinetics, benefiting enzyme replacement therapy with less frequent delivery.^44^ They also boost protein expression, requiring far less RNA for comparable protein levels versus linear modified mRNA. This technology is now being tested for in vivo, scalable vaccine production.^54^, ^55^, ^56^ Additionally, saRNAs can be delivered as two separate transcripts (trans‐amplifying mRNA), reducing overall mRNA size.^57^, ^58^ Exogenous mRNAs can be recognized by Toll‐like receptors (TLRs) and double‐stranded RNA by MDA‐5, which may trigger a moderate to severe immune response.^59^ To prevent immunogenicity, researchers reported that depleting uridine or mixing with pseudouridine during mRNA synthesis is recommended.^60^ In short, the stability of mRNA still needs to be further improved in vivo. In light of the growing importance of stability and safety considerations in the field of gene therapy, vectors are increasingly being used as a means of further investigation. Among them, non‐viral vectors, such as lipid‐based nanoparticles, polymeric nanoparticles, and inorganic nanoparticles, are an emerging class of gene vectors and they offer several advantages. For example, they have improved stability, simple structures, easy large‐scale production, low toxicity, and immunogenicity, and they can accommodate a large number of genes. Among the various types of gene vectors, cationic gene vectors have been extensively studied.^36^

### 3.1 Lipid‐based nanoparticles for mRNA delivery

**FIGURE 3:**
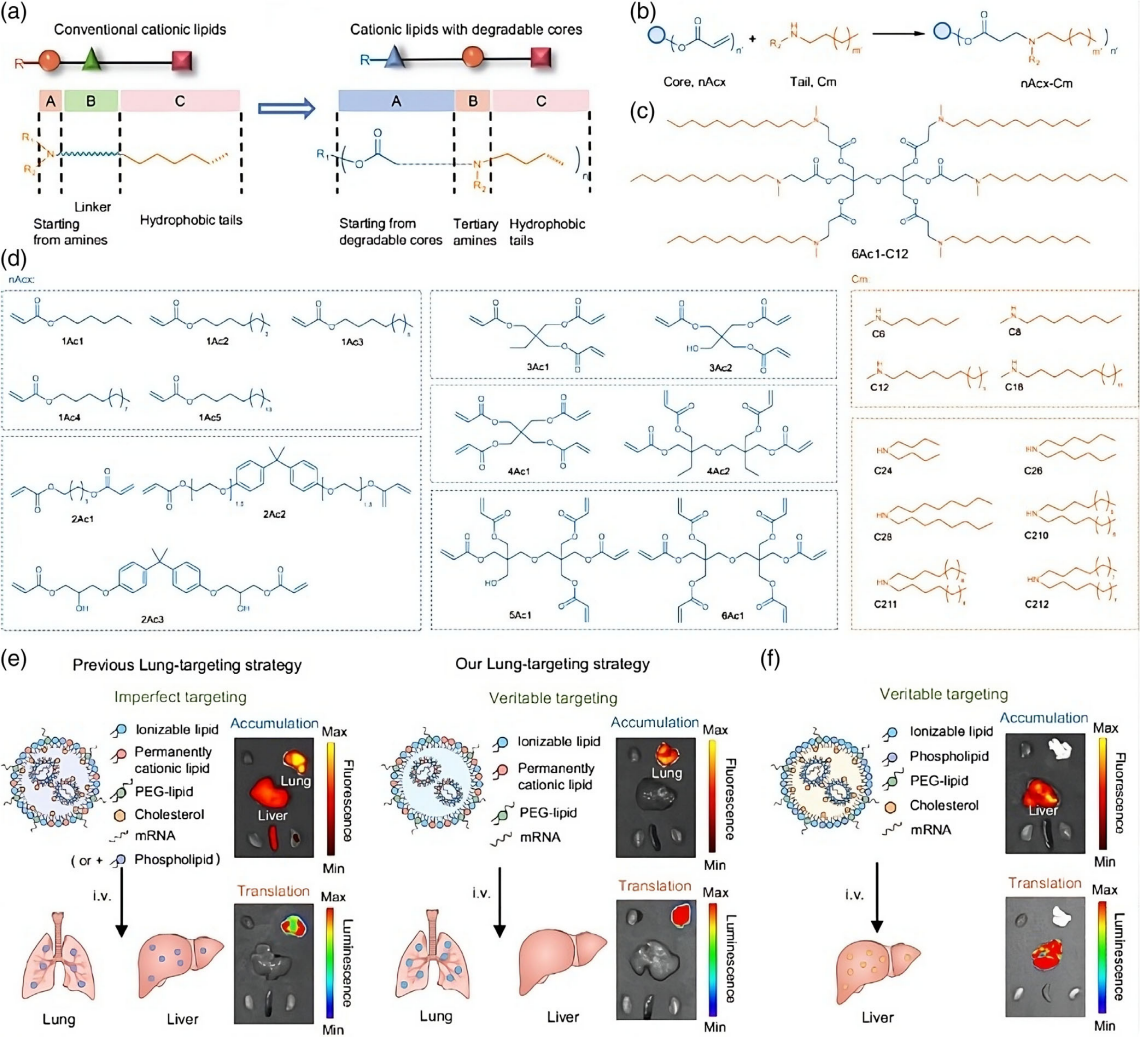
A library of lipids was designed for targeted delivery of mRNA.^67^ (a) A new type of cationic lipid was created with an ester core, and amine tail. (b) The synthesis of nAcx‐Cm lipids involves the Michael addition of degradable ester cores (nAcx) and secondary amines (Cm). In nAcx‐Cm indicates the number of ester bonds in one molecule. (c) The chemical structure of the representative 6Ac1‐C12 lipid is shown. (d) There are 14 degradable cores and 10 hydrophobic amines for lipid synthesis. (e) Schematic of how mRNA accumulates and is translated in the body after being injected. (f) LNPs were altered to target the lungs and liver. Images of mRNA accumulation and translation from 6Ac1‐C12 4‐Comp Lung LNPs (imperfect lung targeting), 6Ac1‐C12 3‐Comp Lung LNPs (veritable lung targeting), and 6Ac1‐C12 Liver LNPs (conventional compositions, veritable liver targeting) were presented.^67^ Copyright © 2024 Springer nature.

In academic studies and clinical trials, lipid‐based nanostructures are often used as non‐viral delivery systems for chemotherapeutic or genetic drugs. Among the classes of nanoparticles used for RNA delivery are lipid‐based nanoparticles, including nanoemulsions (NE), nanocapsules (NC), and lipid nanoparticles (LNP). Among these, LNP is the most widely used. LNP usually consists of four components: an ionizable lipid, cholesterol, a helper phospholipid, and a PEGylated lipid.^61^, ^62^, ^63^, ^64^ These components work together to encapsulate and protect the fragile mRNA. The first mRNA vaccine, mRNA‐1273, used a vector consisting of four lipids, including an ionizable cationic lipid (SM‐102), 1,2‐distearoyl‐sn‐glycero‐3‐phosphocholine (DSPC), cholesterol, and PEG2000‐DMG.^27^ The composition contains neutral auxiliary lipids, which are generally saturated phospholipids that promote the formation and stability of layered lipid structures. Cholesterol has a strong ability to fuse membranes, promoting mRNA internalization and entry into the cytoplasm.^65^ PEG‐functionalized lipids are used for immune escape and increased stability. The ionizable lipids that are most critical remain neutral at physiological pH values to reduce their toxicity and immunogenicity. At low pH values, they carry a positive charge, which allows them to interact with negatively charged nucleic acids through electrostatic forces and achieve lysosomal escape of mRNA after internalization.^66^ For example, Liu Shuai et al. developed a novel LNP delivery technology by removing cholesterol and phospholipids, maintaining the integrity of LNP, and conferring true targeting capabilities (Figure 3).^67^ LNP is designed with a reasonable lipid structure and composition, which enables the simultaneous organ‐targeted accumulation and translation of mRNA. The team has fundamentally altered the conceptualization of LNP, developing 3‐Comp and a conventional LNP based on a central set of biodegradable cationic lipids, thereby attaining precise targeting of the lungs and liver, respectively. In contrast to earlier LNP formulations designed to target liver cells, the customized nAcx CM lipids incorporated into typical LNPs have the potential to induce liver endothelial cell selectivity. Subsequently, the 3‐Comp LNP strategy for removing cholesterol provided a solution to the significant challenge of avoiding liver accumulation and achieving true lung targeting (accumulation and translation) after systemic administration. This organ‐targeting method applies to other existing ionizable cationic lipids and LNPs, thereby enriching the diversity of current LNP delivery systems and expanding their targeting possibilities.^67^ The team's approach to organ‐targeted delivery has demonstrated considerable promise in the development of mRNA drugs with reduced adverse effects. Meanwhile, LNP can be utilized to deliver CRISPR/Cas9 components, enabling genome editing in animal models.^68^ However, the stability of LNP can be affected by interactions between its components. LNP instability can be attributed to physical and chemical factors. To enhance stability and prevent physical instability, measures such as adding polyethylene glycol (PEG) can be taken to prevent aggregation, fusion, and content leakage in LNP. Chemical instability in LNP lipids is mainly caused by hydrolysis and oxidation. For example, DSPC and ionizable cationic lipids are highly susceptible to hydrolysis during storage, which is dependent on temperature and pH due to the presence of carboxylate bonds.^63^, ^69^ When ionizable lipids are present in LNP fractions, aldehyde impurities can be produced through oxidation and hydrolysis pathways. These aldehydes can react with DNA bases and mRNAs, forming covalent adducts that can oxidize encapsulated mRNAs and affect their biological activity.

### 3.2 Polymeric nanoparticles for mRNA delivery

Synthetic or natural cationic polymers (CPs) have received significant attention in the development of gene therapy technology as nucleic acid delivery systems. Examples of commonly used CPs include polyethyleneimine (PEI), polylysine (PLL), and chitosan. CP has a high positive charge density and can interact with negatively charged nucleic acid molecules, compressing them to form small‐sized polyelectrolyte complexes (PICs).^70^ Additionally, it provides a strong pH buffering ability, which facilitates the complex's escape from the body.^71^ PEI, which was first reported in 1995, is one of the most extensively studied cationic carriers for gene delivery in polymer delivery systems. PEI is considered the gold standard for gene transfection due to its high gene encapsulation efficiency in both in vivo and in vitro.^72^ However, the high positive charge density of high‐molecular‐weight PEI can lead to self‐aggregation and adhesion during gene transfer.^73^ To reduce the cytotoxicity of PEI, various methods have been considered in practical applications of gene transfer. One strategy widely used to reduce the cytotoxicity of PEI is to introduce a neutral hydrophilic polyethylene glycol (PEG).^74^ PEG can shield the cationic charge of PEI and reduce non‐specific interactions between complexes and blood components.^74^, ^75^ The synthesis of PEG‐PEI can reduce cell toxicity and prolong blood circulation time in the blood.^75^

**FIGURE 4:**
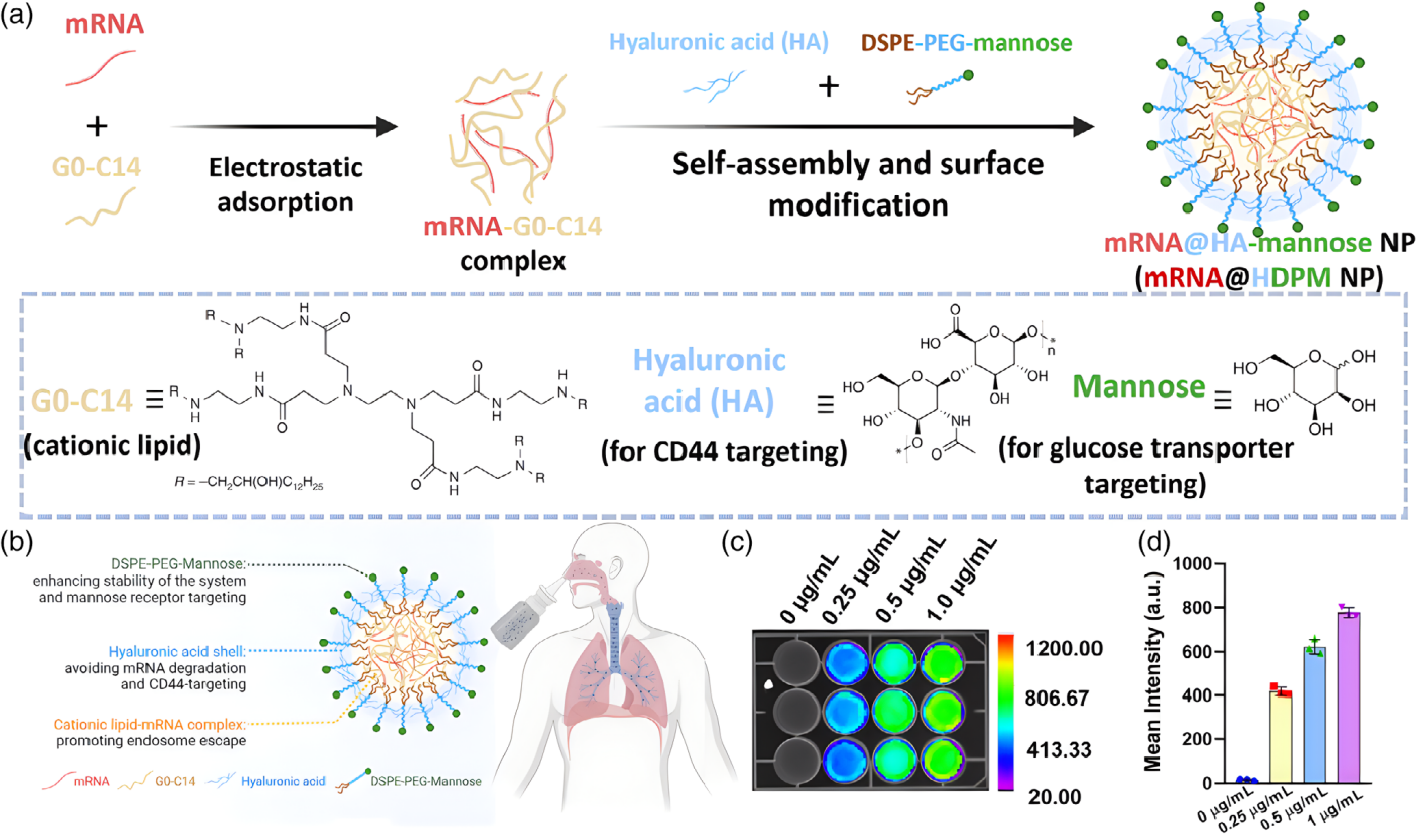
Characterization and Optimization of Dual‐Targeted mRNA Nanoparticles. Schematic illustration of the (a) preparation and (b) inhalation delivery of dual‐targeted mRNA HDPM nanoparticles.^78^ (c) In vitro bioluminescence images and (d) the mean intensity of H1299 cells after treatment with dual‐targeted NPs at different concentrations of Luc‐mRNA.^78^ Copyright © 2023 the PNAS.

In addition to linear polymers, dendritic macromolecules with nanoscale and radial symmetry, such as dendritic polyamide (PAMAM) and polyimide (PPI), are also commonly used as carriers for nucleic acid drugs.^76^ Commonly used cationic polymers (CPs) such as PEI, PDMAEMA, and PLL are non‐degradable and highly cytotoxic, which severely limits their applications. It is widely accepted that the cytotoxicity of CPs is related to their molecular size and positive charge density.^77^ CP with high molecular weight and charge density that is non‐degradable can accumulate in cells, leading to high cytotoxicity. For example, Wei Tao et al. innovatively designed a dual‐targeted mRNA nanoformulation based on CPs and HA, demonstrating ideal stability and efficient transfection of targeted proteins into lung tissue (Figure 4).^78^ More importantly, optimized dual‐targeted mRNA nanoparticles primarily accumulate in lung tumor cells and inflammatory macrophages after inhalation delivery. They also robustly express p53 tumor suppressor, as well as luciferase and green fluorescent protein for imaging, achieving effective lung transfection in vivo.^78^ In addition to self‐assembly between CP and nucleic acid molecules, it is also possible to use pre‐prepared polymer‐based nanomaterials with specific sizes and spatial structures, such as particles, gel, and micelles, to load nucleic acid molecules. This approach can achieve passive targeting, environmental response, slow or controlled release, and other functions.^79^

### 3.3 Inorganic nanoparticles for mRNA delivery

**FIGURE 5:**
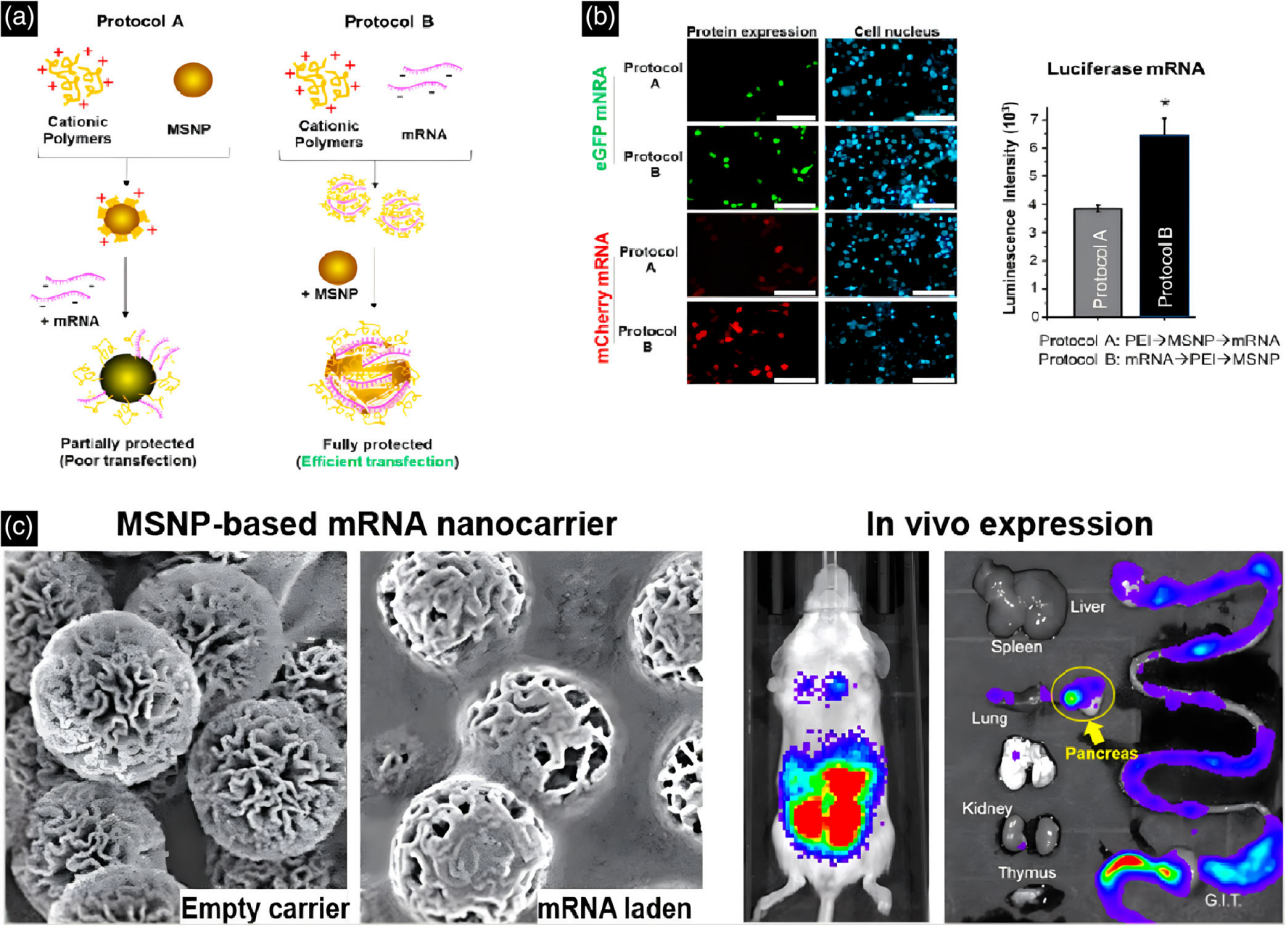
Comparative analysis of mRNA loading protocols using different self‐assembly sequences. (a) Schematic representation of preparing the MSNP and mRNA/PEI‐MSNP mixture using protocol A and B. (b) SEM images of particle. (c) SEM images and IVIS imaging.^91^ Copyright © 2023 American Chemical Society.

Several inorganic nanoparticles have been reported for the delivery and imaging of nucleic acids, including mesoporous silica nanoparticles (MSNP), gold nanoparticles (GNP), and iron oxide nanoparticles (IONP).^80^, ^81^ The carrier is popular due to its ease of synthesis, simple surface modification and functionalization, and high stability and low immunogenicity.^66^ GNP and IONP are generally considered non‐toxic among inorganic nanoparticles.^80^, ^82^, ^83^ GNP is used to deliver nucleic acid molecules through surface modification.^80^ Typically, thiol covalently linked GNP are utilized for DNA and siRNA, which can be directly coupled to gold nuclei or polymer‐modified gold nuclei.^84^, ^85^, ^86^ GNP can be coated with alternating layers of anionic nucleic acids and polymer cations.^80^, ^87^ Targeted ligands can also be added to enable specific interactions and binding with targeted cell surface receptors.^80^, ^88^ Then it is important to consider ligand length, hydrophobicity, and affinity for effective targeting of nanoparticles.^80^, ^89^ IONP, composed of either Fe_3_O_4_ or Fe_2_O_3_, exhibits superparamagnetic properties at specific sizes and can serve as an effective carriers for mRNA delivery.^80^ The majority of delivery systems rely on electrostatic interactions between surface‐modified cationic iron oxide nanoparticles and anionic nucleic acid loads. MSNPs are utilized for nucleic acid delivery because of their excellent biocompatibility and porous structure tunability. Nucleic acid molecules are usually loaded into MSNP through weak non‐covalent interactions.^90^ The loading capacity and nucleic acid release rate are significantly influenced by pore size and surface functionalization.^80^ MSNP with small pore sizes offers adjustable release rates suitable for nucleic acid, whereas MSNP with larger pore sizes provides higher loading capacity and faster release rates.^90^ The loading, protein adsorption, and release rates can be significantly influenced by modifying the negatively charged surface of nanoparticles through cationic surface modification.^90^ For example, Meng Huan et al. discovered a unique assembly method by pre‐mixing mRNA with cationic polymers such as PEI and then binding them to the surface of MSNP through electrostatic interactions, which can significantly improve mRNA transfection efficiency in vitro and vivo.^91^ Due to the potential impact of key physicochemical parameters of MSNP on biological outcomes, the research team further investigated the effects of nanoparticle size, porosity, surface topology, and aspect ratio on mRNA delivery.^91^ Through these efforts, the research team has identified the best performing delivery vector that can achieve efficient cellular uptake and intracellular escape, while effectively delivering mRNA encoding luciferase in mice (Figure 5). Inorganic nanoparticle delivery strategies are expected to improve the biodistribution, delivery efficiency, and safety of mRNA therapeutics. However, inorganic nanoparticles are still in their proof‐of‐concept stages, although the results are promising.

### 3.4 Virus‐like particles

**FIGURE 6:**
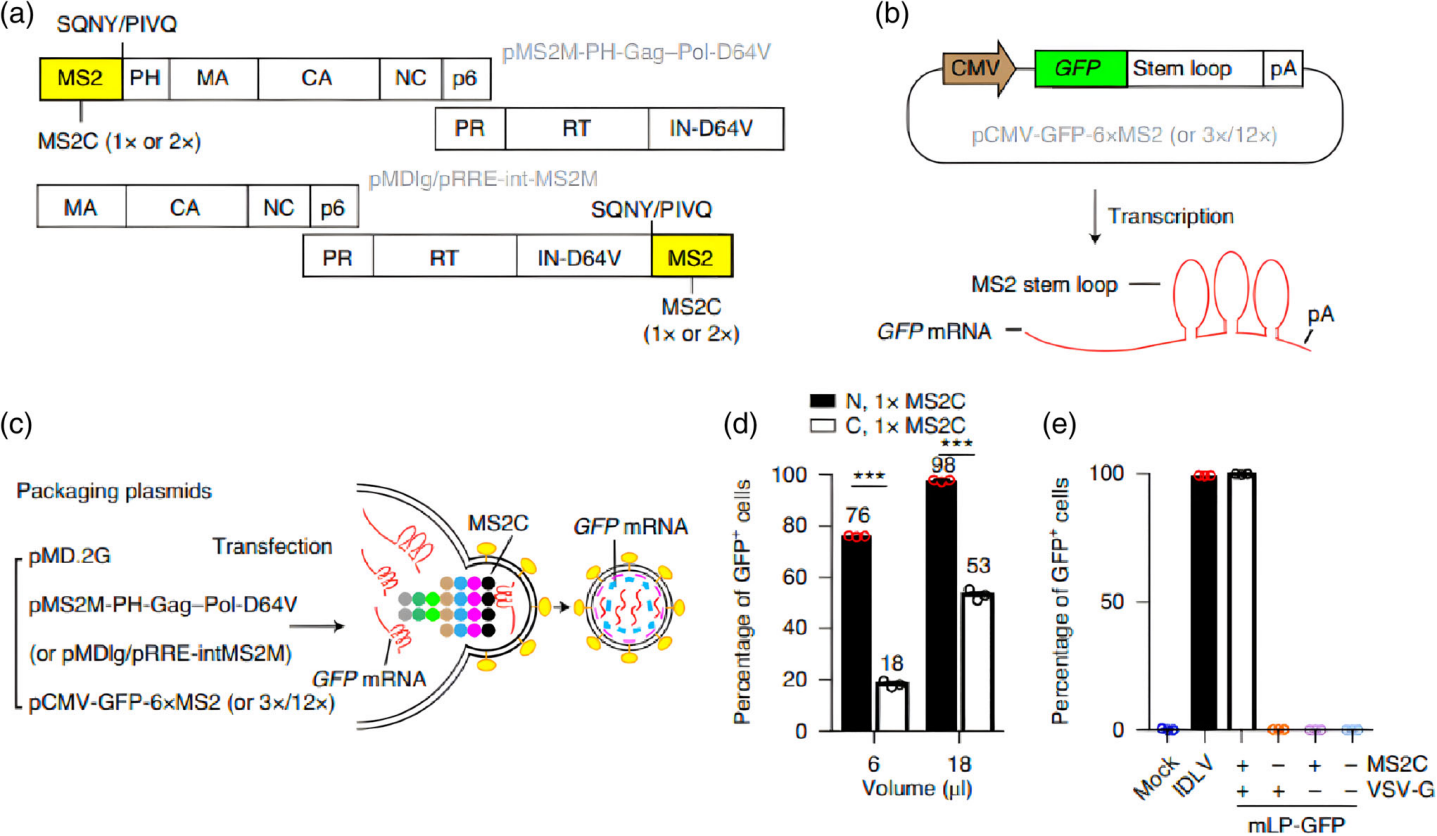
Construction of a lentiviral system for efficient mRNA.^100^ (a) Design of the MS2C‐modified lentiviral Gag‐Pol polyprotein, The MS2C protein was fused to either the N‐terminus or the C‐terminus of Gag‐Pol as a monomer (1×) or dimer (2×). SQNY/PIVQ is an HIV protease cleavage site. Gag is composed of the matrix (MA), capsid (CA), and nucleocapsid (NC), whereas Pol is composed of protease (PR), reverse transcriptase (RT), and integrase which carries a D64V mutation (IND64V). (b) Design of the mRNA‐encoding plasmid encoding the MS2 stem‐loop‐containing GFP mRNA. (c) The principle of mLP‐GFP production. (d) Comparison of mRNA delivery efficiency. (e) The specificity of GFP mRNA packaging to mLP. Copyright © 2024 Springer nature.

In recent years, the development of novel gene delivery modalities has become a popular research direction due to the limitations of viral and nanoparticle‐based gene therapy strategies. Virus‐like particles (VLPs) are a promising alternative as they are viral protein assemblies that can infect cells but lack viral genetic material.^92^ The assembly methods of VLPs can be divided into in vivo assembly and in vitro assembly. The in vivo assembly of VLPs usually refers to the process of assembling one or several types of structural capsid proteins in suitable host cells. When VLPs formed in the body are assembled in the host cells, they may encapsulate relevant components of the host cells into the capsid, which limits the application of such VLPs.^93^ In vitro, assembly generally refers to the process of self‐assembly of capsid proteins outside the cell or in the absence of cells.^94^ The in vitro assembly ability and hollow structure of VLPs can serve as delivery systems for nanoparticles.^95^ In recent years, research on VLPs has mainly focused on production and application.^96^ Among them, VLPs are an excellent choice for delivering DNA, RNA, or pre‐formed ribonucleoproteins into target cells due to their ease of production and low dosage requirement.^97^, ^98^ An engineered virus‐like particle (eVLP) that can efficiently deliver gene editing tools in the form of proteins directly into animal cells has been developed.^99^ Using the principle of virus packaging and specific recognition of messenger RNA stem ring structure and bacteriophage shell protein, the broad‐spectrum recognition of host cells and the characteristics of transient messenger RNA expression are combined simultaneously (Figure 6). Cai et al. has devised an eVLP delivery technology that is capable of transferring genetic material between viral and non‐viral vectors.^100^ They demonstrate lentiviral co‐delivery of Streptococcus pyogenes Cas9 mRNA and vascular endothelial growth factor A (Vegfa)‐targeting guide RNA expression cassettes effectively knocks out Vegfa in a mouse model of wet age‐related macular degeneration.^100^ A single subretinal injection reduced choroidal neovascularization by 63%, without off‐target edits or anti‐Cas9 immune responses.^100^ Engineered lentiviruses for transient nuclease expression may treat retinal neovascular diseases. The results establish eVLP as a promising vector for therapeutic macromolecule delivery, combining the advantages of both viral and non‐viral delivery. However, the use of VLPs in clinical applications is limited due to potential immune reactions to the viral particles.

### 3.5 Exosomes

**FIGURE 7:**
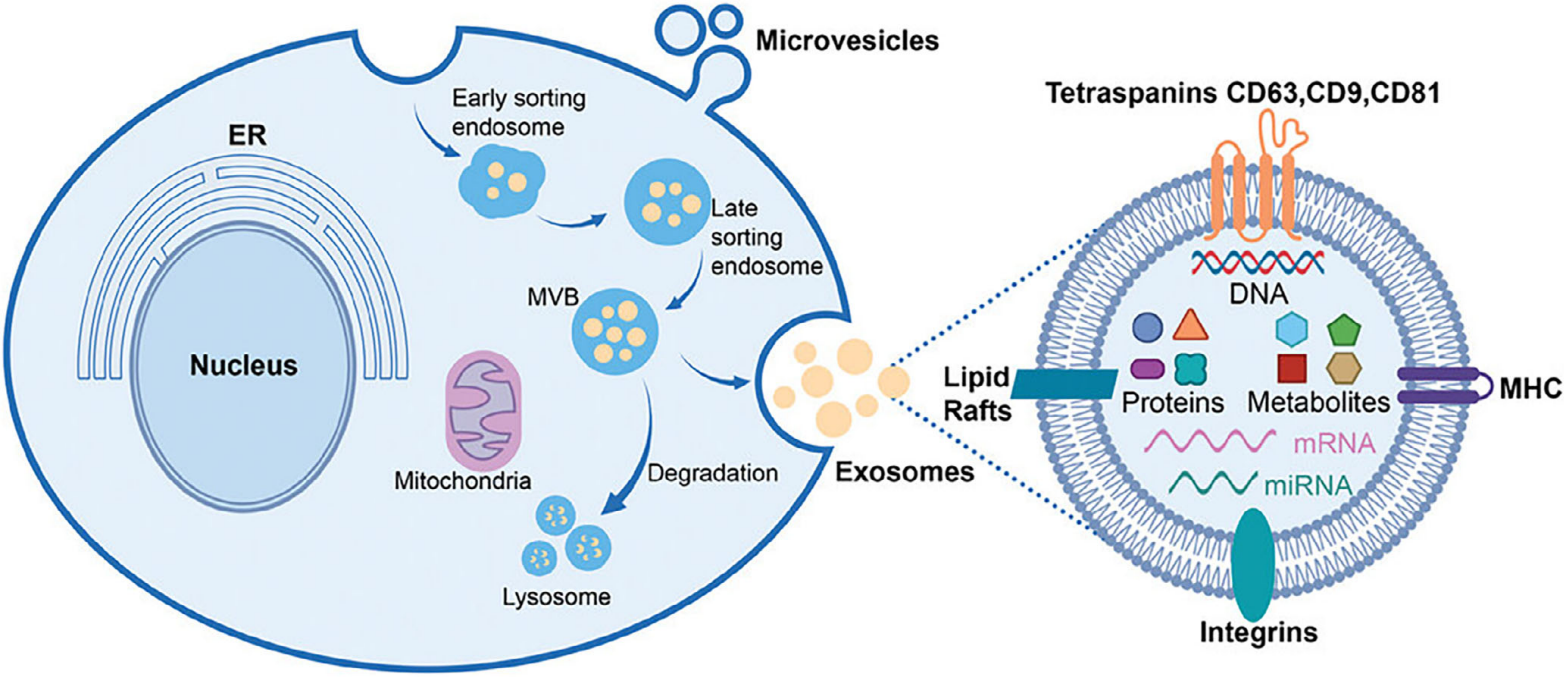
An overview of the biogenesis pathways for two primary types of EVs (exosomes and microvesicles), along with a structural schematic of exosomes. ER, endoplasmic reticulum; MHC, major histocompatibility complex I/II^102^; MVB, multivesicular bodies. Copyright © 2024 Wiley.

Exosomes, extracellular vesicles secreted by cells, have shown promise as carriers for mRNA delivery due to their biocompatibility, pharmacokinetic properties, and ability to penetrate physiological barriers^101^ (Figure 7). Although studies have shown that extracellular vesicles have been proven to be an effective method for messenger RNA delivery in gene therapy, outperforming traditional delivery carriers.^103^, ^104^ However, several limitations exist, including technical difficulties generating exosome–RNA complexes,^105^ limitations with large RNA encapsulation,^106^ and insufficient production from most cellular sources.^107^ Traditional cultures cannot meet clinical demand, requiring weeks‐long incubation to produce sufficient gene‐enriched vesicles.^104^ Currently, two primary methods for loading exosomes with mRNA, including endogenous loading, in which donor cells are engineered to produce and secrete exosomes containing the desired RNA, and exogenous loading, in which purified exosomes from various sources are incubated with the RNA to allow loading.^104^, ^108^, ^109^ Various techniques, such as electroporation and chemical transfection reagents, can be used for exogenous loading. The choice of method depends on several factors, including the type of RNA to be loaded, the quantity required, and the desired target cells. The potential of exosomes as therapeutic and diagnostic agents is rapidly expanding, and further research is needed to optimize loading methods and understand their mechanisms of action. Recently, Yang et al. developed a cellular nanoporation biochip to stimulate cells to secrete exosomes containing therapeutic mRNA.^110^ In glioma mouse models, exosomes containing mRNA were able to restore tumor‐suppressor function, enhance inhibition of tumor growth, and increase survival time. These findings support the potential of therapeutic exosomes. To achieve successful applications of exosome‐based therapeutics, it is important to prioritize the effective release of exosomes by cells, use efficient exosome isolation techniques, and ensure efficient mRNA loading.^111^, ^112^, ^113^

**FIGURE 8:**
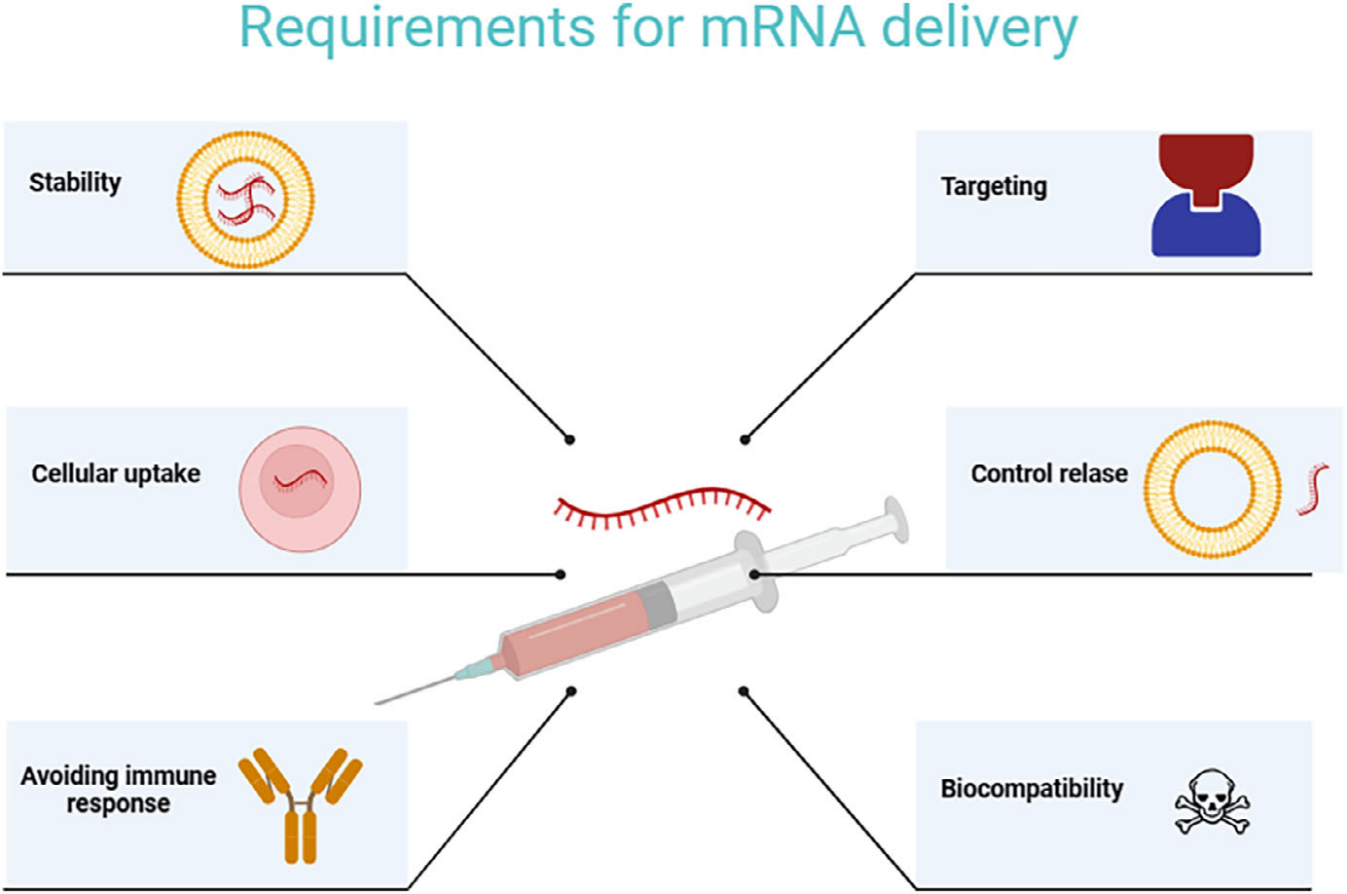
Requirements for mRNA delivery. The delivery system is non‐toxic or has low toxicity. It enhances mRNA stability by encapsulating it, achieves targeted delivery through easily taken‐up carriers, and reduces side effects by precise release inside cells.

The emergence of new strategies is not intended to invalidate traditional methods, but rather to complement them by filling in the gaps in potential applications. It is believed that as technical obstacles are overcome, the potential of mRNA vaccines, other mRNA‐based therapeutic strategies, and the field of gene therapy as a whole will be further realized, ultimately benefiting patients. In summary, a mRNA delivery system is a system used to efficiently deliver mRNA into target cells and ensure its stability, internalization, and correct biodistribution. The following parts are the basic requirements of an mRNA delivery system (Figure 8): (1) mRNA stability: mRNAs are susceptible to degradation in vitro, so the delivery system needs to provide adequate protection against nuclease degradation in the bloodstream or other body fluids.^13^ (2) Facilitate internalization: The delivery system should have an effective cellular uptake mechanism to ensure that sufficient mRNA enters the cell.^12^ (3) Avoiding immune response: The mRNA delivery system should be able to mitigate the immune system's response to prevent the induction of excessive inflammation or other immune responses, thus improving the safety and efficacy of the treatment.^13^, ^114^ (4) Targeted organization or cell targeting: In some cases, it is necessary to ensure mRNA delivery to specific tissues or cell types to maximize therapeutic efficacy and reduce the impact on non‐target tissues.^61^, ^115^ (5) Intracellular release: Once inside the cell, the delivery system should effectively release mRNA, ensuring that it can enter the cytoplasm and participate in related biological processes, such as protein synthesis.^61^, ^65^ (6) Controllability: The system should have a certain degree of controllability to release mRNA when needed, for example, based on changes in the biological environment.^116^, ^117^ (7) Biocompatibility: The components of the delivery system should be biocompatible and not cause serious cytotoxicity or other adverse reactions.

## 4 APPLICATIONS OF MRNA‐BASED THERAPY

Gene therapy involves introducing genetic materials into target cells to correct or compensate for the underlying genetic abnormalities for genetic disorders. In recent years, there has been growing interest in developing new preventive and therapeutic approaches using mRNAs. Some promising applications include vaccine development for infectious diseases and cancer, replacement therapy, and gene editing therapy.

### 4.1 Vaccines for infectious diseases

Since 1989, mRNA has emerged as a novel therapeutic drug concept, offering diverse antigenic determinant.^14^, ^42^, ^118^ Compared with DNA, mRNA do not insert into the genome and are metabolized through normal pathways, ensuring safety.^14^, ^119^ Currently, vaccines for infectious diseases are the most advanced mRNA therapeutic application.^42^ Most mRNA vaccines in preclinical trials and use are administered as a bolus injection into skin, muscle, or subcutaneous space, taken up by immune or non‐immune cells, and translated into antigens for T and B cell display.^42^ By the end of 2019, 15 mRNA vaccine candidates were in clinical trials, with none in phase III trials.^120^, ^121^ It was estimated it would be at least 5–6 years before regulatory approval.^42^ But, the COVID‐19 pandemic overtook the world in 2020, accelerating mRNA vaccine development. For example, As of February 8, 2022, the World Health Organization (WHO) reported 337 COVID‐19 vaccine candidates under development, including 47 mRNA vaccines, 23 of which have entered clinical trials.^122^ The mRNA vaccines Pfizer‐BioNTech (BNT162b2), Moderna (mRNA‐1273), and CureVac (CV7201) were the fastest vaccine developments in history.^122^, ^123^, ^124^, ^125^ The first two received emergency use authorization (EUA) from many regulatory agencies in the United States, United Kingdom, Canada, and Hong Kong, China.^42^, ^122^ On August 23, 2021, the Pfizer‐BioNTech vaccine was the first COVID‐19 vaccine approved for commercialization by the US Food and Drug Administration (FDA).^126^ It was also the first COVID‐19 vaccine approved for children aged 5–11 on October 29, 2021.^122^ It is precisely because mRNA vaccines have obvious advantages in research and development speed, production flexibility, immunogenicity that they have played an important role in epidemic prevention and control. In clinical trials, mRNA vaccines currently have shown high protective efficacy. However, the long‐term safety of mRNA vaccines still needs to be discussed.

### 4.2 Cancer vaccines

A vaccine's core function is to deliver antigens recognized by the immune system to trigger responses.^127^ In developing cancer vaccines, selecting an antigen with high tumor specificity and the ability to induce strong, controllable antitumor T‐cell responses is crucial.^128^ Currently, using combinations of 2–6 shared tumor‐associated antigens (TAAs) has become the trend in targeted mRNA cancer vaccine development.^127^ The selected TAAs are often widely expressed in related tumors and can induce antitumor immune responses when combined with different vectors or adjuvants. To date, hundreds of cancer vaccines have undergone clinical evaluation and three therapeutic cancer vaccines have been approved by the U.S. FDA.^127^, ^129^, ^130^, ^131^ Among them, the first FDA‐approved cancer vaccine was the intravesical bacillus Calmette‐Guerin vaccine (TheraCys) in 1990 for treating and preventing non‐muscle‐invasive urothelial carcinoma after transurethral resection. The prolonged disease‐free survival (DFS) observed in patients with bladder carcinoma in situ (CIS) and Ta/T1 urothelial carcinoma treated with TheraCys was 30 months and 22.5 months, respectively. This compares to a DFS of 4.9 months in CIS patients and 10.5 months in Ta/T1 patients treated with topical doxorubicin. But, factors influencing the development of mRNA cancer vaccines include intrinsic factors of the molecule itself and external factors such as central tolerance to tumor antigens, tumor heterogeneity, and the tumor immune microenvironment. These factors deeply influence the development of mRNA cancer vaccines.

### 4.3 Replacement therapies

Theoretically, mRNAs have the potential to synthesize any type of protein, which could transform the protein manufacturing engine of the cell into a drug factory. This holds great promise for treating a wide range of diseases. However, mRNA replacement therapies have faced challenges in clinical application. For example, in the treatment of hemophilia, where hemophiliacs lack clotting proteins due to a genetic mutation, patients need to be injected with clotting proteins 3–7 times per week due to the short half‐life of the proteins, which is usually only 12 hours.^44^, ^132^ In contrast, preclinical studies in mice have shown that a single weekly injection of a modified linear mRNA at a dose of 0.2–0.5 mg/kg is sufficient to maintain effective clotting protein levels.^133^, ^134^, ^135^ Clinical trials in hemophilia using AAV showed stable protein expression up to 2 years after injection, and recently, the U.S. Food and Drug Administration has approved Roctavian, an AAV‐based gene therapy for the treatment of severe hemophilia A. However, some recent studies have shown that re‐injection may be necessary 5–7 years after injection due to immune system rejection of viral vectors, which have their safety issues, especially in pediatric disease treatment.^44^, ^133^ Developing therapies that target mRNA to specific tissues and provide long‐lasting benefits without excessive side effects are a significant challenge. However, there is only limited progress in this field.

### 4.4 Gene editing therapies

Gene editing is a potent tool for studying gene function and treating genetic diseases. It entails the precise disruption, insertion, or replacement of DNA sequences at specific sites in the genome.^136^ Engineered endonuclease (EEN)‐mediated gene editing has significantly enhanced the efficiency of homologous recombination‐based gene targeting, enabling researchers to edit any gene in various cell types and organisms. For the treatment of transthyretin amyloidosis, sgRNA targeting the disease‐causing TTR gene and mRNA sequences optimized for the spCas9 protein were delivered to the liver via LNP by Gillmore et al.^137^ Clinical data showed that six patients were treated, with three receiving a 0.1 mg/kg dose and three receiving a 0.3 mg/kg dose. After 28 days of treatment, patients who received the two different doses experienced a decrease in plasma species TTR protein levels by an average of 52% and 87%, respectively. At the same time, no serious adverse reactions were observed. In addition to the CRISPR‐Cas9 system, other gene editing tools, such as Base Editing and Prime Editing, can also be delivered in the form of mRNA‐LNP. This delivery method has great potential for inherited diseases. Breda et al. developed CD117/mRNA‐LNP hat encapsulates mRNA and targets stem cell factor receptors (CD117) on hematopoietic stem cells (HSCs).^138^ Based on this technology platform, the delivery of anti‐human CD117/LNP has produced almost complete correction of hematopoietic sickle cells. Furthermore, the in vivo delivery of pro‐apoptotic PUMA (p53 upregulated apoptosis regulator) mRNA and CD117/LNP affects HSC function and allows for non‐genotoxic regulation of Hematopoietic stem cell transplantation (HSCT).^138^ The capacity to target HSCs in vivo provides a non‐genotoxic solution, and this platform may serve as the foundation for in vivo genome editing therapy for hereditary diseases, thereby reducing the necessity for HSCT.

## 5 CONCLUSIONS AND OUTLOOK

In recent years, LNP‐mRNA technology has rapidly developed and revolutionized the fields of medicine and biotechnology, demonstrating great potential for vaccine and gene therapy development. However, it still faces challenges in clinical application. Shorter half‐life and insufficient expression are the major ones. The development of precise delivery systems is a research priority for the future, especially for gene delivery using non‐viral vectors. Overcoming immune barriers is a crucial objective in RNA drug development, as non‐viral vectors can elicit an immune response.^20^ Future research will concentrate on designing vectors that avoid immune responses to ensure RNA drug safety. This may require enhancing the structure of delivery systems and immune escape strategies. Cellular uptake has always been a challenge in the study of non‐viral vectors.^139^ RNA delivery also needs to possess good biodistribution properties and pharmacokinetics to achieve effective therapeutic or preventive effects. As the technology evolves, it is necessary to thoroughly explore mRNA delivery strategies and optimize their designs to overcome these challenges. Innovations in nanotechnology and biomaterials can lead to the design of more efficient mRNA delivery systems, prolong gene expression time, and reduce unwanted side effects, obtaining a good balance between safety and efficacy in mRNA therapeutics.^80^

As CRISPR technology continues to evolve, mRNAs will probably be used more extensively in the field of genetic editing. Personalized therapy will become a crucial direction, with customized mRNA drugs targeting individual genetic profiles for more precise and effective treatments. Additionally, mRNA drugs have multiple functions. First, mRNA can serve as a biomarker for cancer prognosis.^140^ mRNA directly or indirectly affects cellular changes in vivo, resulting in corresponding gene expression and reflecting the pathological state of tissues. Thus, by detecting changes in intracellular mRNA, clues and physiological basis can be provided for early disease detection. Second, mRNA dynamic detection can serve as an effective supplement to tissue testing, with high detection efficiency in asymptomatic populations. It can diagnose and evaluate various diseases, with potential to replace direct tissue sampling detection.^141^ mRNA technology will likely become more widely used in the medical and biomedical fields as technical challenges are overcome, providing strong support for future market applications. It is expected that this progress will bring benefits to human health in the coming years.

## AUTHOR CONTRIBUTIONS

**Qiang Chen:** Writing – original draft; conceptualization. **Ku‐Geng Huo:** Writing – original draft. **Sheng‐Min Ji:** Data curation. **Shu‐De Pang:** Data curation. **Tian‐Ying Sun:** Data curation. **Yi Niu:** Data curation. **Zi‐Hao Jiang:** Data curation. **Peng Zhang:** Writing – original draft; funding acquisition. **Shu‐Xin Han:** Writing – original draft; funding acquisition; writing – review and editing. **Jin‐Yao Li:** Writing – original draft; writing – review and editing; funding acquisition.

## CONFLICT OF INTEREST STATEMENT

All authors declare no competing interest.

## CONSENT FOR PUBLICATION

All authors have provided consent to this publication.

## Data Availability

Data sharing is not applicable to this article as no new data were created or analyzed in this study.
